# The Gut–Heart Axis in Cardiovascular Disease: Microbial Metabolites, Mediterranean and Traditional Turkish Dietary Patterns, and Personalized Medicine

**DOI:** 10.3390/ijms27188311

**Published:** 2026-09-18

**Authors:** Melih Erol Ceyhan, Gentiana Mehmeti, Sude Nazli Cukurova, Şeyma Dümür

**Affiliations:** 1 Faculty of Medicine, Istanbul Atlas University, 34408 Istanbul, Turkey; 35melihceyhan@gmail.com (M.E.C.); sudenazlickrv03@gmail.com (S.N.C.); 2 Faculty of Pharmacy, Ss. Cyril and Methodius University in Skopje, Mother Teresa 47, 1000 Skopje, North Macedonia; genta89m@gmail.com; 3 Department of Medical Biochemistry, Faculty of Medicine, Istanbul Atlas University, 34408 Istanbul, Turkey

**Keywords:** gut–heart axis, gut microbiota, cardiovascular disease, trimethylamine N-oxide (TMAO), short-chain fatty acids (SCFAs), bile acids, Mediterranean diet, traditional Turkish diet, personalized medicine, precision nutrition

## Abstract

Cardiovascular diseases (CVDs) remain the leading cause of morbidity and mortality globally. Increasing evidence identifies the gut microbiota as one of the major regulators of cardiovascular health, leading to the concept of the gut–heart axis. Metabolites from microbiota serve as key mediators linking intestinal microbial activity with vascular and metabolic processes. This comprehensive narrative review was conducted using PubMed and Scopus databases, focusing on studies published between 2015 and 2025, along with selected seminal studies, to show the role of gut microbiota and its metabolites in CVD. Microbial metabolites exert various important and often opposing effects on cardiovascular physiology. Trimethylamine N-oxide (TMAO), derived from dietary choline and carnitine metabolism, promotes atherosclerosis through endothelial dysfunction, inflammation, foam cell formation, impaired cholesterol transport, and enhanced platelet reactivity. In contrast, short-chain fatty acids (SCFAs), produced by microbial fermentation of dietary fibers, exert protective effects by reducing inflammation, improving endothelial function, and maintaining intestinal barrier integrity by G protein coupled receptor activation and histone deacetylase inhibition. Disruption of gut barrier function facilitates lipopolysaccharide translocation, contributing to systemic inflammation and cardiometabolic risk. Bile acids further modulate cardiovascular processes through FXR and TGR5 signaling pathways; this influences lipid metabolism and inflammatory responses. Dietary patterns, including the Mediterranean diet and dietary diversity in Türkiye, play a central role in shaping microbiota composition and metabolite balance. The gut–heart axis represents a complex and dynamic process in which microbiota-derived metabolites exert a critical influence on cardiovascular health and disease. Modulation of the gut microbiome through dietary interventions, microbiota-targeted therapies, and emerging personalized strategies offers promising avenues for the prevention and management of CVDs. Future research should focus on microbiome-based precision approaches and large-scale clinical validation to facilitate translation into clinical practice.

## 1. Introduction

Cardiovascular diseases (CVDs) remain the leading cause of morbidity and mortality worldwide, accounting for approximately 17.9 million deaths annually despite substantial advances in prevention and treatment [1,2]. Among the emerging mechanisms involved in cardiovascular pathophysiology, the gut–heart axis has gained considerable attention over the past decade [3,4]. Growing evidence indicates that the gut microbiota, comprising trillions of microorganisms residing in the gastrointestinal tract, plays a fundamental role in metabolism, immune regulation, and vascular homeostasis [1,2,5]. Consequently, alterations in microbial composition and function have been increasingly implicated in the development and progression of CVD. Dysbiosis, characterized by disruptions in the composition and diversity of the gut microbiota, may impair microbial functions and contribute to CVD pathogenesis [5]. Choline, trimethylamine N-oxide (TMAO), and betaine are three metabolites linked to the dietary lipid phosphatidylcholine. Higher circulating concentrations of these metabolites have been associated with an increased risk of CVD. Studies involving large patient cohorts have demonstrated their prognostic value, while experimental models have shown that choline- or TMAO-enriched diets promote macrophage scavenger receptor expression and accelerate atherosclerotic lesion development [6]. Although numerous reviews have examined individual aspects of the gut–heart axis, few have comprehensively integrated microbial metabolites, intestinal barrier dysfunction, bile acid signaling, Mediterranean and traditional Turkish dietary patterns, and precision medicine within a single cardiovascular framework. This review provides a comprehensive overview of the gut–heart axis by integrating current evidence on microbiota-derived metabolites, intestinal barrier dysfunction, bile acid signaling, Mediterranean and traditional Turkish dietary patterns, and emerging precision medicine strategies for the prevention and management of CVD. Given the breadth of cardiovascular pathophysiology, this review focuses specifically on atherosclerosis and endothelial dysfunction as the mechanistic core of the gut–heart axis, since the majority of the current mechanistic and clinical evidence on microbiota-derived metabolites relates to these processes; while hypertension and other cardiovascular conditions are discussed where directly relevant, a comprehensive treatment of heart failure, arrhythmias, and thrombosis lies beyond the scope of the present review. Specifically, the following sections first examine how individual microbiota-derived pathways TMAO, short-chain fatty acids, and bile acids directly influence vascular and metabolic physiology; they then turn to structural consequences of dysbiosis, namely intestinal barrier dysfunction and endothelial dysfunction, as the convergent mechanistic link between these metabolites and cardiovascular disease; and finally, they consider how dietary patterns shape these microbial pathways and how this knowledge may be translated into therapeutic and personalized medicine strategies.

## 2. Review Methodology

This narrative review was conducted through a structured literature search of the PubMed and Scopus databases for articles published between January 2015 and September 2025. Search terms included combinations of the following keywords, combined using Boolean operators (“AND”/”OR”): “gut microbiota,” “gut–heart axis,” “trimethylamine N-oxide,” “TMAO,” “short-chain fatty acids,” “SCFA,” “bile acids,” “gut dysbiosis,” “Mediterranean diet,” “Turkish diet,” “cardiovascular disease,” and “personalized medicine.” Eligible articles included peer-reviewed original research articles, systematic reviews, and meta-analyses published in English; conference abstracts, non-peer-reviewed sources, and articles without full-text availability were excluded. Titles and abstracts were initially screened for relevance, followed by full-text assessment of potentially eligible articles. In addition, a small number of seminal older studies were included based on their foundational contribution to the field, irrespective of publication date. Given the narrative, non-systematic nature of this review, no formal quality-appraisal tool or PRISMA-based screening flow diagram was applied.

## 3. Choline, Trimethylamine N-Oxide (TMAO), and Betaine

Choline is an essential nutrient abundantly found in foods such as eggs and serves as a major dietary precursor for gut microbiota-mediated trimethylamine N-oxide (TMAO) production [7]. Following dietary intake, choline is metabolized by the gut microbiota into trimethylamine (TMA), which is subsequently oxidized in the liver by flavin-containing monooxygenases, predominantly flavin-containing monooxygenase 3 (FMO3), to generate TMAO [8]. In addition to choline, other dietary substrates, including phosphatidylcholine, L-carnitine, betaine, dimethylglycine, and ergothioneine, contribute to TMAO production through gut microbial metabolism. Circulating TMAO concentrations are influenced by multiple factors, including age, sex, dietary habits, gut microbiota composition, renal function, and hepatic FMO activity [9].

Betaine (trimethylglycine) is a naturally occurring methyl donor that can be synthesized endogenously from choline or obtained directly from dietary sources such as wheat bran, wheat germ, spinach, and beets [10]. Besides its established role in one-carbon metabolism and cellular methylation, a proportion of dietary betaine is metabolized by the gut microbiota into TMA, which is subsequently converted to TMAO in the liver. However, the microbial enzymatic pathways mediating betaine-to-TMA conversion remain less well characterized than the choline-to-TMA pathway catalyzed by the microbial CutC/D enzyme complex, or the analogous carnitine pathway mediated by CntA/B. Proposed routes include direct microbial reduction in betaine via betaine reductase (grdH) or indirect conversion through dimethylglycine decarboxylation, although the relative contribution of each pathway to circulating TMA/TMAO levels in humans has not been firmly established. Consequently, betaine may influence cardiovascular health both directly through methylation-dependent metabolic pathways and indirectly through microbiota-mediated TMAO production, indicating that its cardiovascular effects may not be entirely attributable to TMAO formation.

## 4. Atherosclerosis and TMAO Mechanisms

**Figure 1:**
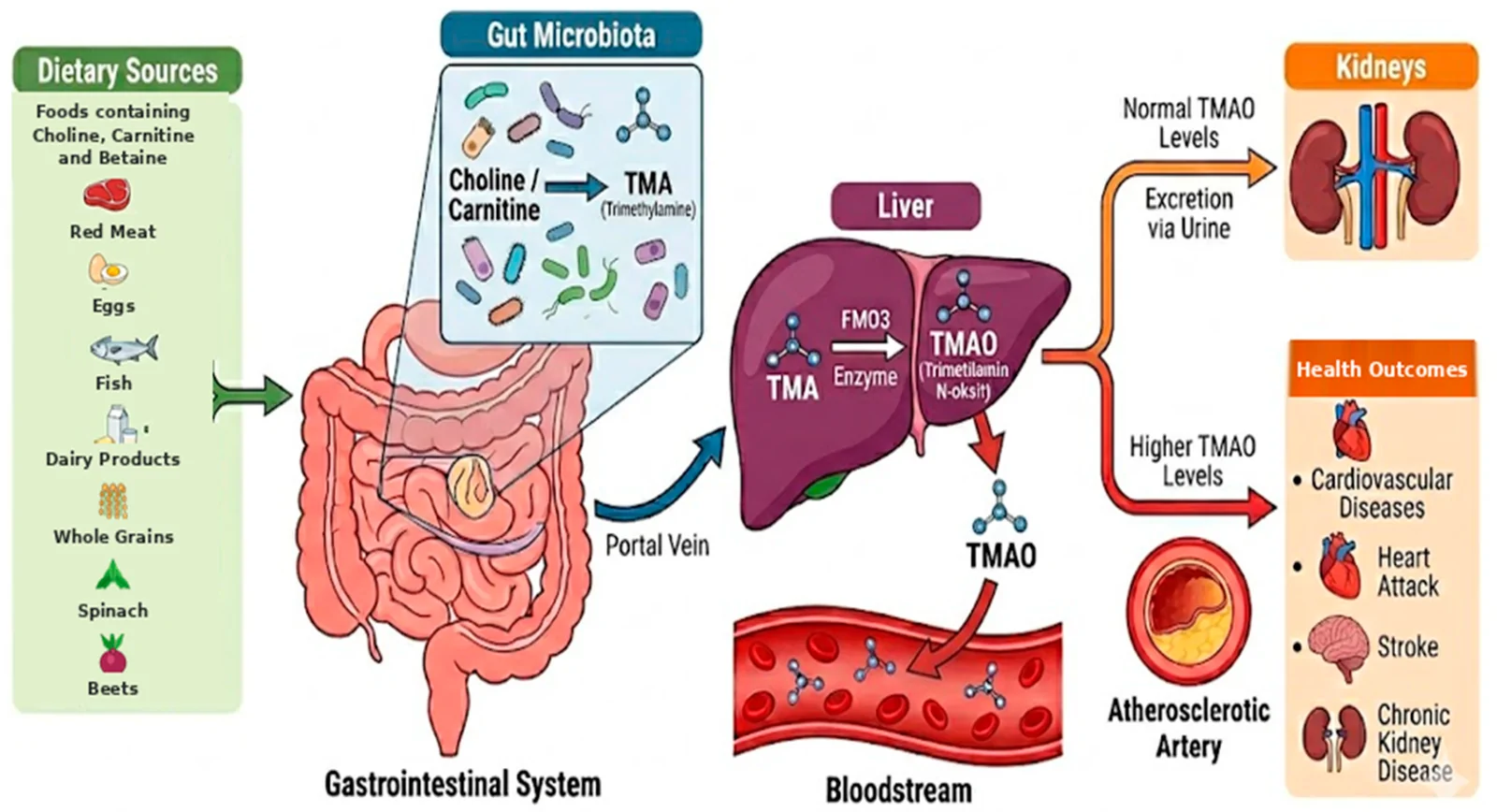
Schematic illustration of trimethylamine N-oxide (TMAO) metabolism and its relationship with the gut microbiota and cardiovascular disease. Dietary nutrients such as choline and L-carnitine are metabolized by the gut microbiota to produce trimethylamine (TMA), which is subsequently converted to TMAO by hepatic flavin-containing monooxygenase 3 (FMO3). Elevated circulating TMAO contributes to endothelial dysfunction, atherosclerosis, thrombosis, and increased cardiovascular risk. Abbreviations: TMA: trimethylamine; TMAO: trimethylamine N-oxide; FMO3: flavin-containing monooxygenase 3. This figure was created by the authors using an AI-assisted illustration tool and is original to this manuscript.

Atherosclerosis is the underlying pathological process responsible for most cases of coronary artery disease (CAD) and many ischemic CVDs. It is characterized by chronic lipid accumulation, inflammation, and fibrotic remodeling within the arterial wall. Progressive plaque formation in the subendothelial space, consisting of lipids, cholesterol, calcium, fibrin, and cellular debris, gradually narrows the arterial lumen and may ultimately result in myocardial infarction, ischemic stroke, or peripheral arterial disease [11,12]. Among gut microbiota-derived metabolites, trimethylamine N-oxide (TMAO) has emerged as a key mediator linking intestinal microbial metabolism to atherosclerosis. Experimental and clinical studies have demonstrated that elevated circulating TMAO levels are associated with an increased risk of adverse cardiovascular events [9,13]. TMAO contributes to atherosclerosis through multiple complementary mechanisms. It promotes vascular inflammation and oxidative stress, leading to endothelial dysfunction and facilitating atheromatous plaque formation [14]. Furthermore, TMAO enhances macrophage activation and foam cell formation, thereby increasing lipid accumulation within the arterial wall and accelerating plaque progression [15,16]. TMAO also suppresses reverse cholesterol transport, limiting cholesterol removal from peripheral tissues to the liver and further promoting atherogenesis [17]. In addition, TMAO enhances platelet hyperreactivity, thereby increasing thrombotic risk [13]. It should be noted that these mechanistic pathways have been demonstrated primarily in experimental and animal models, whereas evidence in humans is largely derived from observational cohort studies showing associations between circulating TMAO levels and cardiovascular outcomes; well-designed clinical trials establishing a causal role for TMAO in human atherosclerosis are currently lacking. Collectively, these findings identify TMAO as a central mechanistic link in the gut–heart axis and suggest that strategies targeting gut microbial TMAO production, including dietary interventions, microbiota modulation, and pharmacological inhibition, may represent promising approaches for the prevention and management of CVD. The pathways linking dietary nutrients, gut microbial metabolism, TMAO production, and CVD are summarized in Figure 1.

## 5. Short-Chain Fatty Acids (SCFAs)

SCFAs are key microbial metabolites produced through the anaerobic fermentation of dietary fibers and indigestible carbohydrates by the gut microbiota in the gastrointestinal tract. They represent one of the most important classes of gut microbiota-derived metabolites involved in regulating immune responses, energy metabolism, intestinal barrier function, and vascular homeostasis. The three major SCFAs; acetate, propionate, and butyrate exert distinct metabolic and immunomodulatory functions, with butyrate serving as the primary energy source for colonocytes and playing a critical role in maintaining intestinal barrier integrity [18,19].

Accumulating evidence suggests that SCFAs contribute substantially to cardiovascular health. In hypertensive patients, reduced intestinal absorption of SCFAs results in increased fecal excretion and lower circulating concentrations. Both experimental and clinical studies have demonstrated that SCFA supplementation can enhance immune function, suppress inflammatory responses, and attenuate cardiovascular complications, including cardiac hypertrophy and fibrosis [18].

At the molecular level, SCFAs exert their biological effects primarily through the activation of G protein–coupled receptors (GPR41 and GPR43) and inhibition of histone deacetylases (HDACs), thereby regulating inflammatory signaling pathways and gene expression. Specifically, GPR41/GPR43 activation suppresses nuclear factor-κB (NF-κB) signaling, thereby reducing the transcription of pro-inflammatory cytokines, while HDAC inhibition promotes histone hyperacetylation and enhances the expression of anti-inflammatory and regulatory genes, including those involved in regulatory T-cell differentiation.

These mechanisms promote immune homeostasis, preserve endothelial function, and maintain vascular integrity [18,20]. Furthermore, SCFAs suppress vascular inflammation by reducing the production of pro-inflammatory cytokines while promoting regulatory immune responses.

Conversely, reduced SCFA production associated with gut dysbiosis may increase intestinal permeability and endotoxemia, thereby promoting vascular inflammation and accelerating atherosclerotic processes [21,22,23]. In addition, SCFAs exhibit anti-atherosclerotic properties by improving endothelial function, reducing inflammation, and modulating lipid metabolism. In contrast to the pro-atherogenic effects of trimethylamine N-oxide (TMAO), SCFAs exert predominantly cardioprotective effects, emphasizing the importance of maintaining a balanced microbial metabolite profile for cardiovascular health [24].

**Figure 2:**
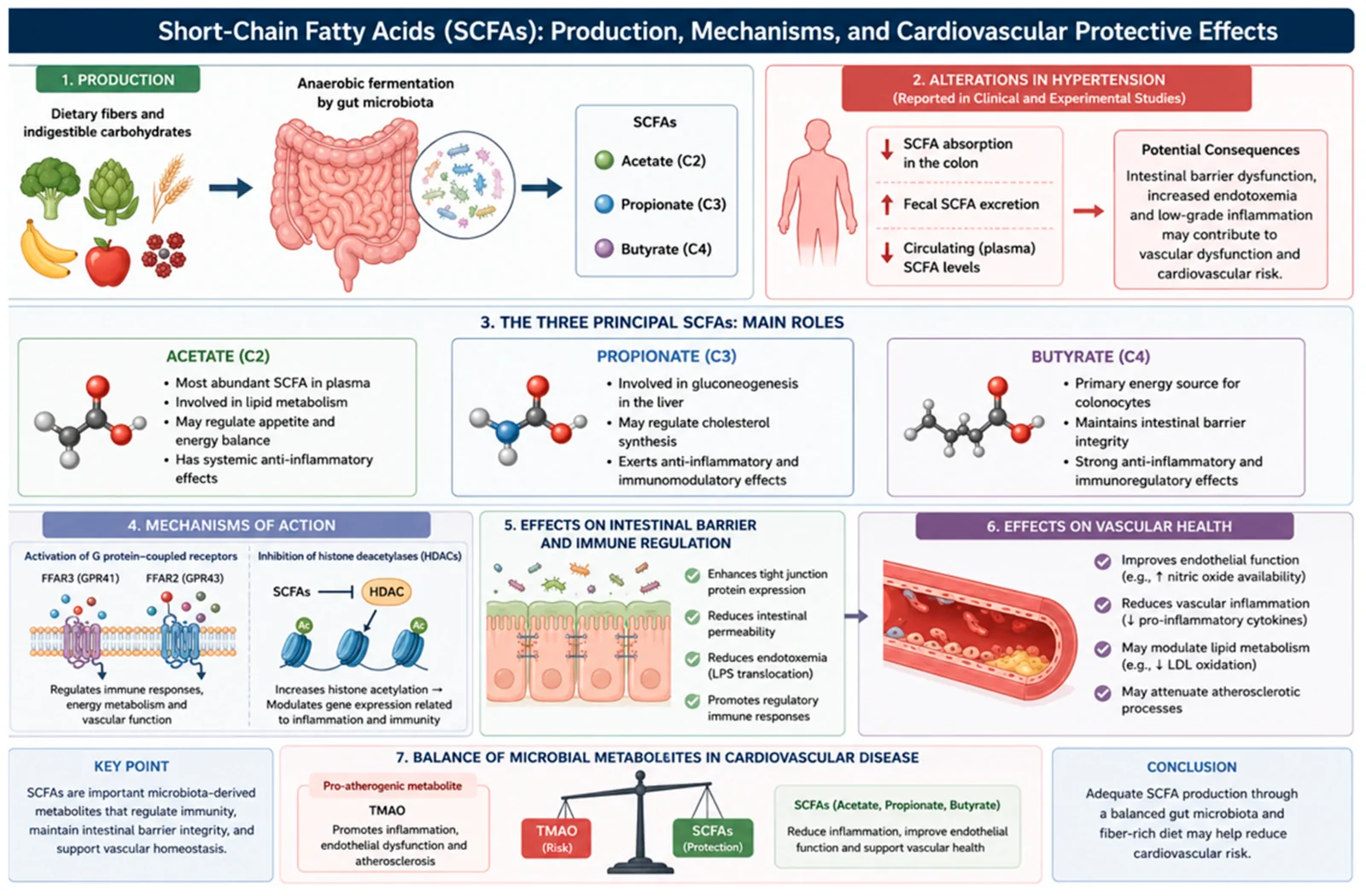
Schematic overview of short-chain fatty acid (SCFA) production and their cardiovascular protective mechanisms. Dietary fibers are fermented by the gut microbiota to produce acetate, propionate, and butyrate. SCFAs regulate immune responses, intestinal barrier integrity, endothelial function, lipid metabolism, and vascular inflammation through activation of G protein–coupled receptors and inhibition of histone deacetylases, thereby contributing to cardiovascular protection. Abbreviations: SCFAs, short-chain fatty acids; GPR43 (FFAR2), free fatty acid receptor 2; GPR41 (FFAR3), free fatty acid receptor 3; HDAC, histone deacetylase; LPS, lipopolysaccharide; TMAO, trimethylamine N-oxide; LDL-C, low-density lipoprotein cholesterol. This figure was created by the authors using an AI-assisted illustration tool and is original to this manuscript.

The production, molecular mechanisms, biological functions, and cardiovascular protective effects of SCFAs are summarized in Figure 2.

## 6. Gut Barrier Dysfunction and Systemic Inflammation

The intestinal barrier plays a pivotal role in maintaining host homeostasis by preventing the translocation of luminal microorganisms and their metabolites into the systemic circulation. The integrity of this barrier depends largely on tight junction proteins, including zonula occludens-1 (ZO-1) and occludin, which regulate paracellular permeability. Disruption of these proteins increases intestinal permeability, a condition commonly referred to as “leaky gut” [25,26]. Impaired intestinal barrier function facilitates the translocation of microbial products, particularly lipopolysaccharide (LPS), into the bloodstream, leading to metabolic endotoxemia [27]. Circulating LPS activates innate immune signaling pathways, primarily through Toll-like receptor 4 (TLR4), triggering the production of pro-inflammatory cytokines such as tumor necrosis factor-α (TNF-α), interleukin-1β (IL-1β), and interleukin-6 (IL-6). Persistent low-grade systemic inflammation contributes to endothelial dysfunction, oxidative stress, and vascular injury, thereby promoting the development and progression of CVDs, including atherosclerosis and hypertension [28]. Emerging evidence suggests that gut barrier dysfunction represents a critical mechanistic link between gut microbiota dysbiosis and cardiovascular pathology. Therefore, strategies aimed at preserving intestinal barrier integrity and reducing endotoxemia may represent promising therapeutic approaches for preventing CVD.

## 7. Bile Acids and Cardiovascular Effects

Bile acids are increasingly recognized as important signaling molecules linking gut microbiota to cardiovascular health. Beyond their classical role in lipid digestion and absorption, bile acids regulate numerous metabolic and inflammatory processes through interactions with nuclear and membrane receptors, particularly the FXR and TGR5 [29,30]. The gut microbiota plays a central role in bile acid metabolism by converting primary bile acids into secondary bile acids, thereby influencing bile acid composition and receptor activation. Alterations in gut microbiota composition may disrupt bile acid homeostasis, leading to dysregulated FXR and TGR5 signaling and contributing to metabolic and cardiovascular disorders [31]. Activation of FXR has been associated with reduced hepatic lipid synthesis and improved cholesterol homeostasis, and regulation of glucose metabolism, whereas TGR5 activation exerts anti-inflammatory and vasoprotective, and metabolic effects through modulation of immune responses and energy expenditure [32]. Consequently, disturbances in bile acid metabolism may promote endothelial dysfunction, chronic inflammation, and atherosclerosis, highlighting bile acids as key mediators within the gut–heart axis [33]. Together with other microbiota-derived metabolites, bile acids constitute an important component of the complex metabolic network linking the gut microbiota to CVD. While TMAO primarily promotes pro-atherogenic pathways and SCFAs exert predominantly anti-inflammatory and vasoprotective effects, bile acids function as metabolic signaling molecules that integrate microbial activity with host lipid metabolism, immune regulation, and vascular homeostasis. The principal mechanisms linking gut microbiota mediated bile acid signaling to cardiovascular health and disease are summarized in Table 1.

## 8. Endothelial Dysfunction as a Key Mechanism of the Gut–Heart Axis

Although TMAO, short-chain fatty acids, and bile acids act through distinct receptors and signaling pathways, a common downstream consequence of these microbiota-derived pathways is endothelial dysfunction, which therefore represents a convergent mechanistic hub linking gut dysbiosis to the vascular pathology underlying cardiovascular disease.

Endothelial dysfunction represents a key mechanism linking gut microbiota dysbiosis to cardiovascular disease. Gut microbiota-derived metabolites exert both detrimental and protective effects on vascular homeostasis. TMAO promotes oxidative stress, impairs nitric oxide bioavailability, and activates inflammatory pathways, leading to endothelial activation and increased expression of adhesion molecules such as VCAM-1 and ICAM-1, thereby facilitating vascular inflammation and atherosclerotic progression [24,27]. Conversely, short-chain fatty acids (SCFAs), particularly butyrate, contribute to endothelial protection by reducing oxidative stress, preserving endothelial barrier integrity, and attenuating inflammatory signaling [28,30]. Moreover, disruption of intestinal barrier integrity facilitates the translocation of lipopolysaccharide (LPS) into the circulation, sustaining chronic low-grade inflammation and endothelial injury [31,33]. These mechanisms collectively contribute to vascular dysfunction and cardiovascular disease progression, as detailed in Table 2. Accordingly, microbiota-directed strategies aimed at restoring gut microbial diversity have demonstrated significant potential for improving vascular and inflammatory outcomes [34]. Therefore, endothelial dysfunction may represent the central biological interface connecting gut dysbiosis, microbial metabolites, systemic inflammation, and cardiovascular pathology. Integration of endothelial biomarkers with microbiome-derived metabolites may further improve cardiovascular risk assessment and support future precision medicine approaches [35,36,37].

## 9. Therapeutic Targeting of the Gut–Heart Axis

Building on the mechanistic pathways described above, this section is deliberately positioned prior to the discussion of specific dietary patterns and precision medicine, as it introduces the general principle of microbiota-directed intervention that subsequent sections on Mediterranean and Turkish dietary patterns, and personalized medicine, will build upon and elaborate in greater depth.

Therapeutic targeting of the gut–heart axis has emerged as a promising strategy for the prevention and management of CVDs. Among the available approaches, dietary modification plays a central role. Increased consumption of dietary fiber promotes the production of short-chain fatty acids (SCFAs), which exert anti-inflammatory, endothelial-protective, and cardiometabolic effects [34]. Conversely, reducing the intake of red meat and choline- and L-carnitine-rich foods may decrease the production of trimethylamine N-oxide (TMAO), thereby lowering pro-atherogenic risk [3]. In addition to dietary intervention, microbiota-directed therapies, including probiotics, prebiotics, and synbiotics, have demonstrated potential for restoring gut microbial diversity and improving metabolic, inflammatory and vascular outcomes [34]. Pharmacological strategies targeting microbial metabolism are also gaining increasing attention. For example, inhibitors of microbial TMA production, such as 3,3-dimethyl-1-butanol (DMB), have been shown to reduce circulating TMAO levels and attenuate atherosclerosis in experimental models. It should be noted that DMB and related TMA-lyase inhibitors remain at the preclinical stage, with their efficacy and safety in humans yet to be established. Likewise, modulation of bile acid signaling through FXR and TGR5 agonists represents a promising approach for improving lipid metabolism and suppressing inflammation [24]. Emerging therapeutic strategies, including fecal microbiota transplantation (FMT) and microbiome-based precision medicine, aim to directly modify gut microbial composition and function to improve cardiovascular health [34]. However, FMT and microbiome-based precision medicine remain largely experimental in the cardiovascular context, with evidence currently limited to small pilot studies and extrapolation from other disease areas; these approaches should therefore be regarded as investigational rather than clinically validated interventions at this stage, in contrast to dietary modification, which has stronger and more consistent clinical support. However, several challenges remain, including interindividual variability in gut microbiota composition, uncertainties regarding long-term safety, and the limited availability of large-scale clinical trials validating these interventions. Despite these limitations, therapeutic modulation of the gut–heart axis represents a rapidly evolving field with considerable potential to complement conventional cardiovascular therapies and advance precision medicine approaches for CVD prevention and management. Future studies should focus on well-designed randomized clinical trials to establish the efficacy, safety, and long-term cardiovascular benefits of microbiota-targeted interventions.

## 10. Mediterranean Diet and the Gut–Heart Axis

The Mediterranean diet is one of the most extensively studied dietary patterns associated with cardiovascular protection and beneficial modulation of the gut microbiota. It is characterized by a high intake of fruits, vegetables, whole grains, legumes, nuts, and seeds, with olive oil serving as the primary source of dietary fat. Moderate consumption of fish and dairy products, together with limited intake of red and processed meats, further distinguishes this dietary pattern. Numerous epidemiological studies randomized clinical trials and meta analyses have consistently demonstrated that adherence to the Mediterranean diet is associated with a lower incidence of cardiovascular events and improved cardiometabolic health [35]. One of the key mechanisms underlying these benefits is the modulation of gut microbiota composition and function. The high dietary fiber content promotes the growth of beneficial microbial taxa and enhances the production of SCFAs, including acetate, propionate, and butyrate. These metabolites maintain intestinal barrier integrity, regulate immune responses, and reduce systemic inflammation. By preserving tight junction proteins and preventing increased intestinal permeability, SCFAs limit the translocation of lipopolysaccharide (LPS), into the circulation, thereby reducing metabolic endotoxemia and its downstream cardiovascular consequences [36]. The Mediterranean diet also reduces exposure to dietary precursors of TMAO. Lower consumption of red meat and choline- and L-carnitine-rich foods decreases substrate availability for microbial TMA production while favoring a gut microbial composition less enriched in TMA-producing bacteria. Consequently, this dietary pattern promotes a more favorable balance between pro-atherogenic metabolites, such as TMAO, and cardioprotective metabolites, including SCFAs [37]. In addition, the Mediterranean diet is rich in bioactive compounds, including polyphenols, flavonoids, and monounsaturated fatty acids derived primarily from olive oil. These compounds exhibit antioxidant and anti-inflammatory properties, improve endothelial function, and beneficially modulate gut microbiota composition. Polyphenols, in particular, have been shown to exert prebiotic-like effects by increasing microbial diversity and stimulating the growth of beneficial bacterial species [35,38]. Collectively, these mechanisms demonstrate that the Mediterranean diet exerts its cardioprotective effects through integrated modulation of the gut–heart axis. By enhancing beneficial microbial metabolites, reducing pro-atherogenic pathways, preserving intestinal barrier integrity, and attenuating systemic inflammation, the Mediterranean diet represents a cornerstone non-pharmacological strategy for the prevention and management of CVD.

## 11. Traditional vs. Modern Turkish Dietary Patterns and the Gut–Heart Axis

The traditional Turkish diet shares several characteristics with the Mediterranean dietary pattern, including high consumption of vegetables, legumes, fermented dairy products such as yogurt, and the use of olive oil, particularly in coastal regions. These dietary components have been associated with beneficial modulation of the gut microbiota, enhanced production of SCFAs, and preservation of intestinal barrier integrity [35]. However, dietary habits in Türkiye have changed substantially over recent decades, with a progressive shift toward a more Westernized dietary pattern characterized by increased consumption of refined carbohydrates, processed foods, excessive salt, and red and processed meats [39]. Bread remains a staple food in Türkiye and is a major contributor to daily sodium intake, which has been associated with alterations in gut microbiota composition, impaired vascular function, and increased cardiovascular risk [40]. The increased consumption of red meat and other animal-derived foods also provides greater amounts of choline and L-carnitine, the principal dietary precursors of trimethylamine TMAO. As discussed previously, elevated circulating TMAO concentrations have been associated with endothelial dysfunction, chronic inflammation, platelet hyperreactivity, and accelerated atherosclerotic progression [41]. At the same time, lower consumption of dietary fiber may reduce SCFA production, impair intestinal barrier integrity, and promote metabolic endotoxemia and systemic inflammation. Collectively, these dietary changes may contribute to gut microbiota dysbiosis and disrupt the balance between cardioprotective and pro-atherogenic microbial metabolites. Although many traditional components of the Turkish diet retain considerable cardioprotective potential, preserving these dietary habits while limiting the adoption of Westernized eating patterns may represent an effective strategy for modulating the gut–heart axis and reducing cardiovascular risk in the Turkish population. In contrast to these Westernizing trends, several traditional dietary components continue to offer cardioprotective potential.

The traditional Turkish diet includes several components with the potential to promote cardiovascular health through beneficial modulation of the gut microbiota. Fermented dairy products, particularly yogurt and kefir, provide beneficial microorganisms and bioactive compounds that support microbial diversity, intestinal barrier integrity and gut homeostasis [42]. Legumes and bulgur, which are staple foods in traditional Turkish cuisine, are rich sources of dietary fiber and resistant starch. Their fermentation by the gut microbiota enhances the production of SCFAs, particularly butyrate, thereby supporting intestinal barrier function, regulating immune responses, and reducing systemic inflammation [43,44]. Traditional vegetable dishes prepared with olive oil combine fiber-rich plant foods with monounsaturated fatty acids and polyphenols, which exhibit antioxidant and anti-inflammatory properties while improving endothelial function and cardiometabolic health. In addition, traditionally fermented foods, such as tarhana and yogurt, may further promote microbial diversity and contribute to maintaining gut microbial homeostasis [44,45].

Collectively, these traditional dietary components may enhance the production of beneficial microbial metabolites, preserve intestinal barrier integrity, and attenuate systemic inflammation, thereby contributing to cardiovascular protection through modulation of the gut–heart axis. The principal dietary components shared by the Mediterranean and traditional Turkish dietary patterns, together with their potential effects on gut microbiota composition, microbial metabolism, and cardiovascular health, are summarized in Table 3.

## 12. Personalized Medicine and the Gut Microbiome in Cardiovascular Disease

Advances in microbiome research and multi-omics technologies have accelerated the development of personalized approaches for the prevention and management of the CVD. The gut microbiota exhibits substantial inter-individual variability influenced by genetics background, dietary habits, environmental exposures, medication use, and lifestyle factors [46,47]. This variability affects the production of microbial metabolites, including TMAO and SCFAs, thereby contributing to differences in individual cardiovascular risk. Personalized nutrition represents one of the most promising applications of microbiome-based medicine. Dietary interventions tailored to an individual’s gut microbiota composition may enhance the production of beneficial metabolites, such as SCFAs while reducing the generation of pro-atherogenic metabolites, including TMAO. For example, individuals with a microbiota profile enriched in TMA-producing bacteria may benefit from targeted reducing dietary intake of choline and carnitine-rich foods, whereas individuals with reduced SCFA-producing capacity may benefitfrom increased dietary fiber intake or targeted prebiotic supplementation [48]. Microbiome profiling using metagenomics, metabolomics, and other multi-omics approaches, enables comprehensive characterization of microbial composition and function. These technologies facilitate the identification of microbial signatures associated with cardiovascular risk and may improve early disease detection, risk stratification and therapeutic monitoring [49]. In addition, circulating microbial metabolites, particularly TMAO, are being investigated as potential biomarkers for guiding individualized therapeutic strategies and evaluating treatment response [50]. The integration of microbiome-derived data with conventional cardiovascular risk factors represents a key component of precision cardiology [51]. By combining microbiome profiling with clinical, genetic, and metabolic information, more accurate risk prediction models and individualized therapeutic strategies may be developed. Emerging interventions, including targeted probiotics, microbiota-directed therapies, and precision nutrition, further support the transition toward personalized cardiovascular care. Although challenges remain including methodological heterogeneity, limited standardization, and the need for large-scale prospective clinical studies, microbiome-based precision medicine holds considerable promise for improving cardiovascular prevention, risk assessment, and patient outcomes.

## 13. Future Perspectives and Current Challenges

Despite growing body of evidence supporting the role of the gut–heart axis in CVD, several challenges continue to limit the translation of microbiome-based discoveries into routine clinical practice. The gut microbiota is a highly complex and dynamic ecosystem influenced by dietary habits, age, genetics, medication use, environmental exposures and geographical location. This substantial inter-individual variability complicates the identification of universal microbial signatures and limits the development of standardized therapeutic strategies [52]. Most current evidence has been derived from experimental, observational, and relatively small clinical studies, making it difficult to establish causal relationships between specific microbial alterations, microbiota-derived metabolites, and cardiovascular outcomes [4,51,53]. Furthermore, methodological heterogeneity in sample collection, sequencing technologies, metabolite quantification, and bioinformatics pipelines complicates comparisons across studies and reduces reproducibility [54,55]. Although microbial metabolites such as TMAO and SCFAs have shown considerable promise as biomarkers of cardiovascular risk, their clinical interpretation may be influenced by renal function, dietary intake, age, medication use, and other metabolic factors [24,56]. Currently, no universally accepted clinical cut-off or risk-stratification threshold has been established for circulating TMAO; most reported associations are based on relative differences between quartiles or tertiles of TMAO concentration across study populations rather than on validated absolute thresholds. Consequently, standardized methodologies and large-scale prospective studies are required to validate these biomarkers and determine their clinical utility. Advances in microbiome profiling, metagenomics, metabolomics, and other multi-omics technologies are expected to accelerate the development of precision cardiovascular medicine. Future research should focus on integrating microbiome-derived data with clinical, genetic, and metabolic information to improve cardiovascular risk prediction and enable individualized therapeutic strategies. In parallel, well-designed randomized controlled trials are needed to evaluate the long-term efficacy and safety of microbiota-targeted interventions, including dietary modification, probiotics, prebiotics, synbiotics, fecal microbiota transplantation, and pharmacological modulation of microbial metabolism [52,57]. Collectively, overcoming these scientific and clinical challenges will be essential for translating current knowledge of the gut–heart axis into evidence-based strategies for CVD prevention and management. Another relatively underexplored dimension of the gut–heart axis is the endocrine-like capacity of the gut microbiota, which can synthesize and modulate hormones and neurohormones including catecholamines, serotonin, GABA, and steroid hormones such as estrogens with potential downstream effects on vascular tone, blood pressure regulation, and sex-specific cardiovascular risk. Future studies integrating this hormonal axis with established metabolite pathways such as TMAO and SCFA signaling may provide a more complete mechanistic picture of the gut–heart axis [58].

## 14. Conclusions

The gut–heart axis represents a complex bidirectional communication network linking the gut microbiota with cardiovascular health and disease. Throughout this review, we have highlighted how microbiota-derived metabolites, including TMAO, SCFAs, and bile acids, together with intestinal barrier integrity and systemic inflammation, contribute to cardiovascular pathophysiology. Dietary patterns play a central role in shaping the gut microbiota and its metabolic activity. While the Mediterranean and traditional Turkish dietary patterns promote the production of beneficial microbial metabolites and support intestinal barrier function, the increasing adoption of Westernized dietary habits may favor gut dysbiosis, enhance pro-atherogenic pathways, and increase cardiovascular risk. Emerging microbiota-targeted therapies, advances in microbiome profiling, and precision medicine approaches provide exciting opportunities for individualized cardiovascular prevention and treatment. However, substantial challenges remain, including interindividual variability, methodological heterogeneity, and the need for large-scale clinical validation. A deeper understanding of host-microbiota interactions will be essential for translating microbiome science into clinical practice. As research continues to evolve, modulation of the gut–heart axis has the potential to become an integral component of future precision cardiovascular medicine.

## Figures and Tables

**Table 1:** Major mechanisms linking gut microbiota-mediated bile acid signaling to cardiovascular health and disease.

Bile Acid Related Process	Gut Microbiota Interaction	Main Receptor	Biological Effects	Cardiovascular Implications
Primary bile acid metabolism	Gut microbiota converts primary bile acids into secondary bile acids	FXR	Regulates lipid and glucose metabolism	Maintains cholesterol homeostasis and metabolic balance [29,30]
Secondary bile acid formation	Microbial deconjugation and biotransformation modify the bile acid pool	FXR and TGR5	Modulates metabolic and inflammatory signaling pathways	Preserves vascular homeostasis and reduces inflammation [31]
FXR activation	Gut microbiota influences the composition of FXR ligands	FXR	Reduces hepatic lipid synthesis, regulates bile acid synthesis, and improves cholesterol metabolism	Attenuates atherogenic lipid accumulation and metabolic dysfunction [32]
TGR5 activation	Secondary bile acids activate membrane receptor signaling	TGR5	Suppresses inflammatory responses, enhances energy expenditure, and improves glucose metabolism	Improves endothelial function and vascular protection [32]
Bile acid dysregulation	Gut dysbiosis disrupts bile acid composition and receptor signaling	Impaired FXR/TGR5 signaling	Promotes chronic inflammation and metabolic imbalance	Contributes to endothelial dysfunction, atherosclerosis, and CVD [33]

Note: The mechanisms summarized above are supported by a combination of mechanistic, preclinical (animal/cell model), and, in some cases, clinical observational studies; the relative strength of evidence varies across pathways and warrants further validation in humans.

**Table 2:** Endothelial biomarkers associated with the gut–heart axis.

Biomarker	Clinical Significance
VCAM-1	Endothelial activation and atherosclerosis; upregulated (↑) by TMAO-induced oxidative stress and inflammatory signaling, reflecting gut dysbiosis-driven vascular injury [21,24,27].
ICAM-1	Vascular inflammation; increased (↑) alongside VCAM-1 in response to TMAO and endotoxemia-related endothelial activation [21,24,27].
Angiopoietin-2	Endothelial permeability; elevated (↑) as an emerging marker linking microbiome-derived inflammatory signaling to microvascular destabilization [35,36,37].
von Willebrand factor	Endothelial injury and thrombosis; increased (↑) in association with TMAO-related platelet hyperreactivity and endothelial damage [13,35,36,37].

Abbreviations: VCAM-1: vascular cell adhesion molecule-1; ICAM-1: intercellular adhesion molecule-1. ↑, increased/upregulated.

**Table 3:** Key components of the Mediterranean and traditional Turkish dietary patterns and their potential effects on the gut–heart axis.

Dietary Component	Representative Mediterranean and Traditional Turkish Sources	Effects on Gut Microbiota	Microbial/Metabolic Effects	Potential Cardiovascular Effects
Dietary fiber	Legumes, whole grains, vegetables, fruits	Promotes microbial diversity and SCFA-producing bacteria	↑ SCFA production	Reduced inflammation and improved endothelial function
Polyphenols	Extra-virgin olive oil, grapes, pomegranate, herbs, tea	Supports beneficial microbial populations	Modulates microbial metabolites and antioxidant pathways	Reduced oxidative stress and vascular inflammation
Unsaturated fatty acids	Extra-virgin olive oil, nuts, seeds, fish	Favors a healthier microbial profile	Improves lipid metabolism	Improved cholesterol homeostasis and reduced cardiovascular risk
Fermented foods	Yogurt, kefir, ayran, tarhana	Provides beneficial microorganisms and supports microbial diversity	Supports intestinal barrier integrity	Reduced systemic inflammation
Traditional plant-based foods	Lentils, chickpeas, beans, bulgur, vegetables	Enhances microbial diversity and saccharolytic fermentation	↑ SCFA production and ↓ potentially pro-atherogenic microbial metabolites	Improved metabolic and vascular homeostasis

Abbreviations: SCFA, short-chain fatty acid. Note: Among the listed sources, extra-virgin olive oil, grapes, and pomegranate are characteristic of the broader Mediterranean diet, whereas kefir, ayran, tarhana, and bulgur are specific to traditional Turkish cuisine; legumes, whole grains, vegetables, fruits, nuts, seeds, fish, and yogurt are shared components of both dietary patterns. ↑, increased/upregulated. ↓ decreased/downregulated.

## Data Availability

No new data were created or analyzed in this study. Data sharing is not applicable to this article.

## Abbreviations

The following abbreviations are used in this manuscript:

CAD	Coronary artery disease
CVDs	Cardiovascular diseases
DMB	3,3-Dimethyl-1-butanol
FFAR2	Free fatty acid receptor 2
FFAR3	Free fatty acid receptor 3
FMT	Fecal microbiota transplantation
FMO3	Flavin-containing monooxygenase 3
FXR	Farnesoid X receptor
GPR41	G protein-coupled receptor 41
GPR43	G protein-coupled receptor 43
HDAC	Histone deacetylase
ICAM-1	Intercellular adhesion molecule-1
IL-1β	Interleukin-1 beta
IL-6	Interleukin-6
LDL-C	Low-density lipoprotein cholesterol
LPS	Lipopolysaccharide
SCFAs	Short-chain fatty acids
TGR5	Takeda G protein-coupled bile acid receptor 1
TLR4	Toll-like receptor 4
TMA	Trimethylamine
TMAO	Trimethylamine N-oxide
TNF-α	Tumor necrosis factor-alpha
VCAM-1	Vascular cell adhesion molecule-1
ZO-1	Zonula occludens-1
